# Detection of Genetically Altered Copper Levels in *Drosophila* Tissues by Synchrotron X-Ray Fluorescence Microscopy

**DOI:** 10.1371/journal.pone.0026867

**Published:** 2011-10-28

**Authors:** Jessica C. Lye, Joab E. C. Hwang, David Paterson, Martin D. de Jonge, Daryl L. Howard, Richard Burke

**Affiliations:** 1 School of Biological Sciences, Monash University, Melbourne, Victoria, Australia; 2 X-ray Fluorescence Microscopy, Australian Synchrotron, Melbourne, Victoria, Australia; University of Medicine and Dentistry of New Jersey, United States of America

## Abstract

Tissue-specific manipulation of known copper transport genes in *Drosophila* tissues results in phenotypes that are presumably due to an alteration in copper levels in the targeted cells. However direct confirmation of this has to date been technically challenging. Measures of cellular copper content such as expression levels of copper-responsive genes or cuproenzyme activity levels, while useful, are indirect. First-generation copper-sensitive fluorophores show promise but currently lack the sensitivity required to detect subtle changes in copper levels. Moreover such techniques do not provide information regarding other relevant biometals such as zinc or iron. Traditional techniques for measuring elemental composition such as inductively coupled plasma mass spectroscopy are not sensitive enough for use with the small tissue amounts available in *Drosophila* research. Here we present synchrotron x-ray fluorescence microscopy analysis of two different *Drosophila* tissues, the larval wing imaginal disc, and sectioned adult fly heads and show that this technique can be used to detect changes in tissue copper levels caused by targeted manipulation of known copper homeostasis genes.

## Introduction

Copper is an essential nutrient required in trace amounts by most organisms. The main cellular use of copper is as a co-factor for vital copper-dependent enzymes (cuproenzymes) such as Cu, Zn superoxide dismutase, lysyl oxidase, tyrosinase and peptidylglycine α-amidating monooxygenase [1], [2], [3]. Cellular copper uptake requires the activity of members of the Ctr1 family of transmembrane proteins which are located at the outer plasma membrane [4], [5]. The copper-translocating P-Type ATPase proteins ATP7A (Menkes, MNK) and ATP7B (Wilson Disease, WND) are then required for copper transport across the *trans* Golgi Network membrane into the Golgi lumen for incorporation into cuproenzymes such as tyrosinase [6], [7]. Copper is toxic when present in excess in the cell, catalysing the generation of free oxygen radicals that result in damage to lipids, proteins and DNA [2], [8]. ATP7A/B can respond to elevated copper levels by translocating to the outer plasma membrane (PM) to export copper from the cell [9], [10]. Thus copper homeostasis is a balance of copper uptake (Ctr1) and efflux (ATP7A/B) as well as sequestration within the cytoplasm by metallothionein proteins.

The vinegar fly *Drosophila melanogaster* is an excellent model for studying the mechanisms of copper homeostasis *in vivo* [11]. Copper transport mechanisms are conserved between vertebrates and insects with *Drosophila* having two major *Ctr* genes, *Ctr1A* and *Ctr1B*, required for baseline and inducible copper uptake respectively [12], [13], [14]. A single *ATP7* gene, *DmATP7* is required for copper efflux [15]. Ctr1A and B are observed on the apical outer membrane of polarized cell types such as midgut enterocytes whereas DmATP7 is seen on the basolateral membrane [16], [17], consistent with a directed flow of copper transport from the midgut lumen, through the enterocytes and out into the circulating lymph. The power of *Drosophila* as a system for discovering novel copper homeostasis genes is highlighted by the recent identification via genetic screen of the requirement for the vSNARE gene *Syntaxin5* for optimal copper accumulation [18].

The GAL4/UAS system is a bipartite system used to target genetic manipulations to specific tissues or cell types in *Drosophila* [19]. Genes can be either ectopically expressed using UAS cDNA transgenes, or suppressed by way of UAS RNA interference (RNAi) transgenes. This technique has been used in *Drosophila* to show that decreased uptake and/or increased efflux of copper causes a loss of adult cuticle pigmentation and in extreme cases, loss of sensory bristles and a cleft in the adult thorax [15], [20]. The developing eye is also affected, with decreased copper uptake resulting in a smaller, flattened, misshapen eye [20]. Excess copper, caused by increased uptake and/or decreased efflux also causes dramatic thoracic and eye phenotypes and can even cause pupal lethality in extreme circumstances [20], [21], [22].

It has been assumed that the phenotypes reported are due to alterations in cellular copper levels, consistent with the known action of Ctr1 and ATP7 proteins in other species and in cultured cells. However a method for directly measuring copper levels in small tissues is currently lacking. Supporting evidence has come from studies using the copper-responsive transgenes *Ctr1B_EYFP* and *MtnB_EYFP* [12], however these reporters are not active in all tissues and may also be responsive to changes in other metals (*MtnB* for instance is also induced by increased zinc levels). Copper-sensitive fluorophores show considerable promise [23] but the first-generation copper sensors lack sufficient sensitivity to detect subtle changes in elemental levels. Spectroscopy techniques such as inductively coupled plasma (ICP) mass spectroscopy (MS), atomic absorption spectroscopy (AAS) and atomic emission spectroscopy (AES) are powerful methods for quantifying elemental levels but require relatively large amounts of tissue and do not provide topological information.

X-ray fluorescence microscopy (XFM) combined with synchrotron radiation can be used to generate x-ray fluorescence (XRF) elemental maps of biological tissues [24], [25], [26], [27], [28]. Here, this technique is employed in an elemental analysis of two different *Drosophila* tissues - whole-mount larval imaginal discs, and microtome sections of adult fly heads - to detect changes in cellular copper levels resulting from targeted manipulation of known copper transport genes. The technique is shown to be effective in detecting subtle changes in metal distribution within tissues and will be useful for further studies of novel candidate metal homeostasis genes.

## Methods

### *Drosophila* Maintenance and Stocks

All *Drosophila* strains and crosses were maintained on standard medium at 25°C unless stated otherwise. *w^1118^* (BL3605, Bloomington Stock Centre, Indiana USA). *GMR-GAL4*, *P{GMR-GAL4.w^−^}2* (BL9146). *PNR-GAL4*, *P{GawB}pnr^MD237^* (BL3039). RNA interference (RNAi) lines obtained from the Vienna *Drosophila* RNAi Centre include: V8315 (*DmATP7*), V46757 and V46758 (*Ctr1A*), V5804 and V5805 (*Ctr1B*) [29]. Over expression lines *pUAST DmATP7*, *pUAST dNCtr1A*, and *pUAST Ctr1B* have been described previously [15], [20].

### Sample preparation

Wandering third instar larvae were dissected in cold PBS then fixed in 4% Paraformaldehyde in PBS for 30 minutes. Fixed tissues were washed in PBS then rinsed briefly in H_2_0 prior to two final 2-minute washes in 0.1 M ammonium acetate. Imaginal discs were transferred to a silicon nitride window (Silson Ltd: SiRN-7.5-200-3.0-500) along with a small drop of ammonium acetate. The remaining liquid and tissues were left to air dry overnight at room temperature.

Heads were removed from three day old flies and fixed overnight in 10% neutral-buffered formalin (Grale Scientific) on a rotating wheel. After a 6 hour paraffin processing cycle the heads were individually embedded into 6 mm×6 mm paraffin moulds. Heads were sectioned on a rotary microtome (Microtec® *4060*) to a thickness of 9 µm. Sections were placed onto silicon nitride windows and Superfrost® Plus glass slides (Lomb Scientific Pty Ltd.) and air dried for 1 hour at room temperature. Sections were then incubated at 60°C for 40 minutes, to allow the paraffin to partially melt. The remaining paraffin was removed by four 2-minute washes in absolute xylene (Ajax Finechem Pty Ltd). The silicon nitride windows were air dried for 15 minutes post-washing. Superfrost glass slides were stained with a haemotoxylin-eosin stain.

### X-ray fluorescence analysis

X-ray fluorescence (XRF) elemental maps were collected at the X-ray Fluorescence Microscopy (XFM) beamline at the Australian Synchrotron. The beamline uses an undulator source, a pair of Si(111) crystals, and a Kirkpatrick-Baez mirror pair to form a focus of monochromatic x-rays with spot size around 2×2 µm [30], [31]. For these studies x-rays of 9.9 keV energy were used to excite K-shell fluorescence emission from the first row transition metals, i.e., elements Sc to Zn. Secondary x-ray fluorescence and scattering was collected using a 96-element prototype Maia detector [32], which was oriented at 90° to the beam at a distance of 20 mm. The Maia detector system uses real-time event-mode processing by means of a massively-parallel on-board FPGA, and streams x-ray events (energy, time-over-threshold, and detector identity) to computer disk during acquisition. Interleaved with this stream are the sample stage locations, updated every 1 µm of stage transit in both the horizontal and vertical directions. The samples were raster scanned with constant horizontal velocity motion (‘on the fly’) combined with vertical steps of 1 µm resulting in elemental maps with 1 µm square pixels. The horizontal sample stage was scanned at velocities between 0.05 and 2.0 mm/s, resulting in a pixel transit (or dwell) times as short as ∼0.5 ms with minimal overheads. This dwell time is two to three orders of magnitude shorter than that routinely used with conventional x-ray fluorescence detectors and allowed the collection of large numbers of high definition elemental maps in few-hour time periods.

The XRF event stream was analysed using GeoPIXE [33], [34]. This software uses Dynamic Analysis to subtract background and resolve overlapping peaks when generating elemental maps, thus allowing calculation of semi-quantitative values for all the different elements. Elemental quantification is achieved primarily by using an ab-initio model of x-ray interactions [35], verified and calibrated using standard reference metal (Mn, Pt) foils and multi-element standards [36]. The detected x-ray signals in each pixel are related to calculated x-ray yields for an assumed homogenous organic tissue composition with uniform section thickness of 30 µm. Initial images had a striped appearance due to sample stage velocity artefacts which were later corrected by normalizing to the elastic scatter signal.

Beyond visual comparison of elemental maps, a standard Pearson colocalization analysis was investigated by use of ‘scatter plots’ of the quantitative concentrations of two elements. Highlighting the spatial locations of distinct populations identified within the scatter plots led to the discovery of significant structural variation between several phenotypes.

## Results

### Ectopic expression of copper uptake genes alone or in combination with suppression of a copper efflux gene causes detectable increases in tissue copper levels

**Figure 1:**
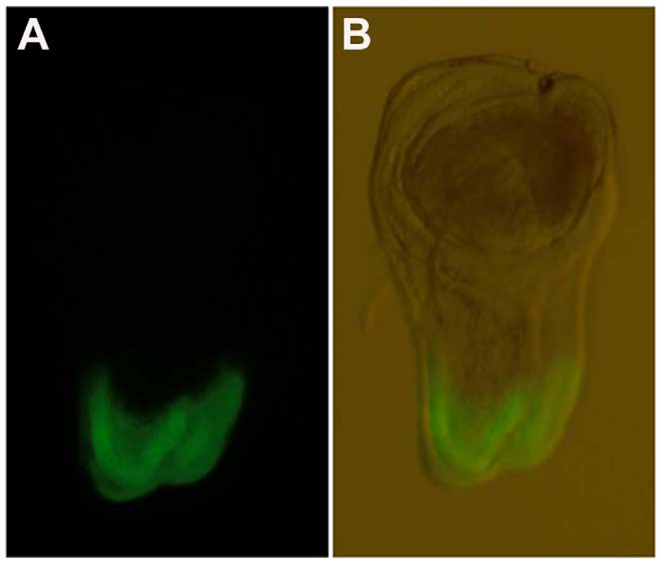
*Pannier-GAL4* drives expression in the dorsal tip of the wing imaginal disc. Wild-type third instar wing imaginal disc showing GFP (green) under the control of the *pannier-GAL4* driver. In all wing discs shown, anterior is to the left and ventral is to the top. Right panel shows same disc under white light to visualize disc structures. *pannier-GAL4* is expressed exclusively in the dorsal-most region of the disc, as seen by the GFP expression.

XRF elemental maps were collected for wing imaginal discs dissected from wandering third instar *Drosophila* larvae. This tissue is the larval precursor of the adult fly wing and thorax and was chosen for analysis because it is an epithelial monolayer, just one cell thick. Furthermore using the GAL4/UAS system, genetic manipulations can be carried out specifically in a subsection of this tissue, allowing the remaining, un-manipulated tissue to be used as a control region for comparison. Figure 1A shows a typical mature wing imaginal disc with Green Fluorescent Protein (GFP) signal in the dorsal-most quarter of the disc showing the expression domain of the *pannier-GAL4* driver used in subsequent experiments.

**Figure 2:**
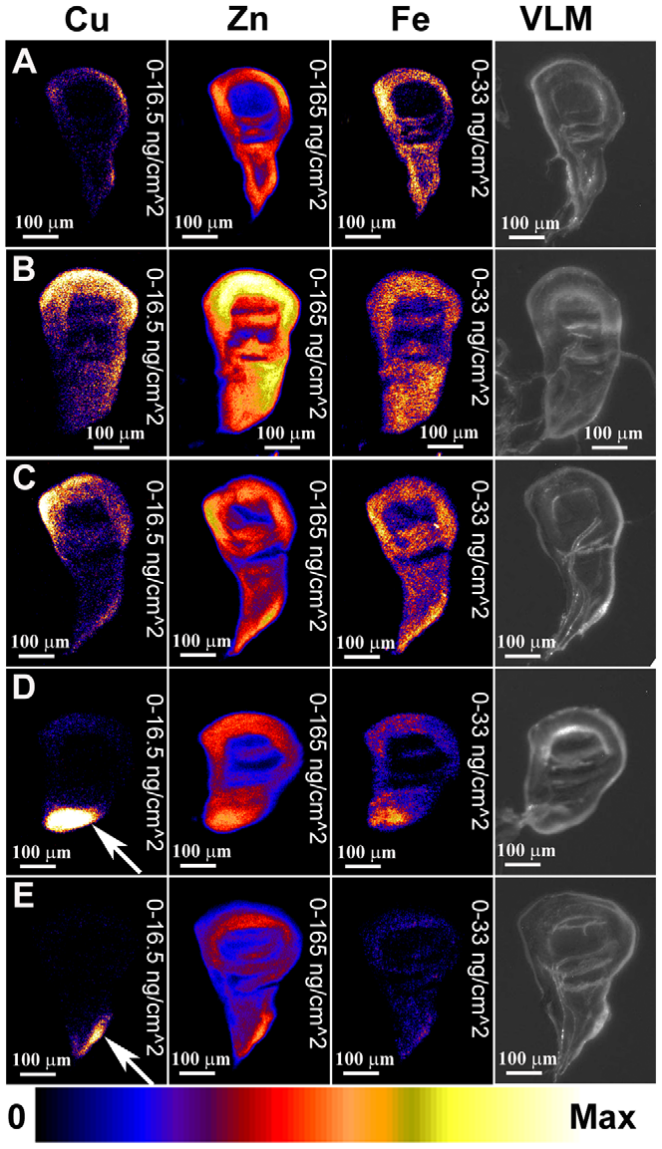
Manipulation of copper transport genes results in altered copper distribution detected by XRF. XRF analysis of third instar wing imaginal discs, showing distribution of copper (left panel), zinc (middle panel) and iron (right panel). Distribution is shown as a heat map with the relative concentration shown at the bottom of the figure. For all imaginal discs shown in Figures 2, 3, 6, S1, S2 and S3 element quantification is normalised so that maximum values are as follows: copper - 16.5 ng/cm^2^, zinc – 165 ng/cm^2^, iron - 33 ng/cm^2^. In the far right panels a visible light microscopy (VLM) image is shown for each disc. A) Wild-type control disc. B) One copy of *dNCtr1A-FLAG* under *pannier-GAL4* control. No change in copper distribution relative to zinc is seen. C) Two copies of *dNCtr1A-FLAG* under *pannier-GAL4* control. No change in copper distribution relative to zinc is seen. D) One copy of *dNCtr1A-FLAG* together with one copy of *Ctr1B-FLAG* under *pannier-GAL4* control. Dramatic increase in copper relative to zinc is seen in the dorsal *pannier* domain (arrow). E) One copy of *dNCtr1A-FLAG* together with one copy of *DmATP7 RNAi* under *pannier-GAL4* control. Strong increase in copper relative to zinc is seen in the dorsal *pannier* domain (arrow).

Analysis of several wild-type wing imaginal discs showed a relatively even distribution of the three major elements detected, copper, zinc and iron (Figure 2A). Some accumulation of copper in the margin of the discs, particularly ventrally, was observed in comparison to iron and zinc. Zinc was the most abundant of the first-row transition metals, followed by iron, and then copper. Copper levels detected were in some cases close to the limits of detection for these sample sections and using these relatively fast scanning parameters.

Next, the effect of increasing copper uptake by ectopic expression of a *UAS dNCtr1A-FLAG* construct under control of the *pannier-GAL4* driver was assessed. No changes in relative copper levels were observed in the dorsal *pannier* domain of the disc, either with one (Figure 2B) or two (Figure 2C) copies of the *dNCtr1A* transgene. Previous studies have shown this transgene to have low copper uptake activity – it can rescue the copper deficiency phenotype caused by *DmATP7* over expression [15] but has no over expression phenotype when expressed alone [20], unlike a similar *Ctr1A* transgene lacking the C-terminal FLAG epitope , which causes lethality when over expressed in the fly [21].

Combination of the *UAS dNCtr1A-FLAG* transgene together with a *UAS Ctr1B-FLAG* transgene causes a dramatic rough-eye phenotype when these constructs are ectopically expressed in the developing eye [20]. This combination phenotype is much more severe than the mild rough-eye phenotype produced by *UAS Ctr1B-FLAG* alone [20]. When this transgene combination was tested by XRF, a dramatic increase in copper levels was seen in the *pannier* domain compared to the remainder of the wing disc (Figure 2D, seen in 5/5 discs of this genotype). Likewise, combination of the *UAS dNCtr1A-FLAG* transgene together with a *UAS DmATP7 RNAi* transgene also causes a severe rough-eye phenotype [20] and also results in greatly elevated copper levels in the *pannier* domain of the wing disc (Figure 2E, seen in 4/4 discs of this genotype). In each case of elevated copper levels, zinc levels remained unperturbed whereas iron levels were sometimes elevated but not to the extent of the increase in copper levels. Therefore strong increase in copper uptake or mild increase in copper uptake combined with suppression of copper efflux both result in: 1) strong visible phenotypes in the adult fly; and 2) clear increases in copper levels in the larval tissues that give rise to adult structures.

**Figure 3:**
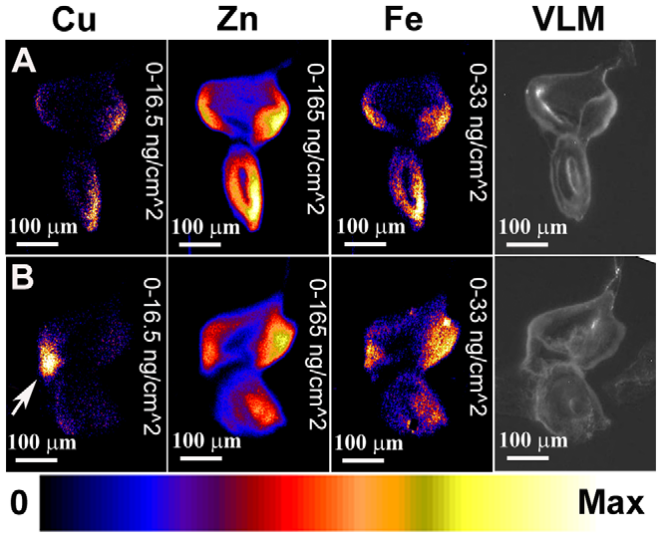
XRF also detects altered copper distribution in eye imaginal discs. XRF analysis of third instar eye-antennal imaginal discs, showing distribution of copper (left panel), zinc (middle panel) and iron (right panel). Dorsal is to the left, posterior to the top. Far-right panel shows a visible light microscopy (VLM) image of each disc. A) Wild-type control disc. B) One copy of *dNCtr1A-FLAG* together with one copy of *Ctr1B-FLAG* under *pannier-GAL4* control. Dramatic increase in copper relative to zinc is seen in the dorsal *pannier* domain (arrow).

To confirm that this result is general and not confined to the wing imaginal disc, eye imaginal discs were also analysed. Like the wing imaginal discs, wild-type eye imaginal discs displayed a relatively even distribution of copper, zinc and iron, with a consistent accumulation of all three elements near the ventral margins on the discs probably due to the tissue being thicker in this region (Figure 3A). The *pannier-GAL4* driver is also active in the eye imaginal discs and analysis of eye discs from *pannier-GAL4*/*UAS dNCtr1A-FLAG*+*UAS Ctr1B-FLAG* flies showed an additional accumulation of copper only in the dorsal side of the disc corresponding to the *pannier* domain in these tissues (Figure 3B, arrow). Elemental maps of additional wing and eye discs of the same genotypes seen in Figures 2 and 3 are shown in Figures S1 and S2 for comparison.

### Visible shifts in copper distribution are confirmed by Pearson colocalization analysis

**Figure 4:**
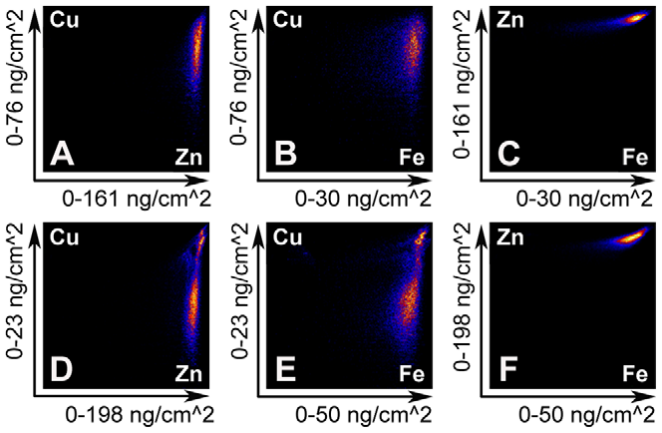
Colocalization analysis of wildtype and manipulated wing imaginal discs. Standard Pearson colocalization analysis of two of wing imaginal discs. A–C) Analysis of the wild-type disc from Figure 2A. D–F) Analysis of the *pannier-GAL4*/*UAS dNCtr1A*+*UAS Ctr1B* disc from Figure S1G. For each of the two discs, colocalizations between copper and zinc (A and D), copper and iron (B and E) and zinc and iron (C and F) are shown as scatter plots, with the maximum level of each element in ng/cm^2^ shown on the X and Y axes. The wild-type disc shows a tight association between zinc and iron (C) and a broader, unimodal association between copper and zinc (A) and between copper and iron (B). The *pannier-GAL4*/*UAS dNCtr1A*+*UAS Ctr1B* disc also shows a tight unimodal association between zinc and iron (F) but the associations between copper and zinc (D) and between copper and iron (E) are both broader than wild-type and clearly bimodal, with a separate, high relative-copper cluster present in both plots.

Pearson colocalization analysis was used to investigate shifts in copper distribution relative to other elements. GeoPIXE allows the co-distribution of element pairs to be represented graphically as a scatter plot. Figure 4 shows the associations between copper and zinc (Figure 4A), copper and iron (Figure 4B) and zinc and iron (Figure 4C) for the wild-type wing disc shown in Figure 2A. Populations identified within the scatter plot can be isolated and their spatial distribution visualised by highlighting them within the elemental maps. There is a tight association between zinc and iron whereas copper varies considerably with respect to both zinc and iron, resulting in a single long, narrow cluster. In contrast to wild-type discs, colocalization analysis of *pannier-GAL4*/*UAS dNCtr1A-FLAG*+*UAS Ctr1B-FLAG* wing discs showed clear bimodal associations between both copper and zinc (Figure 4D) and copper and iron (Figure 4E) whereas zinc and iron remained tightly associated in a single cluster (Figure 4F).

**Figure 5:**
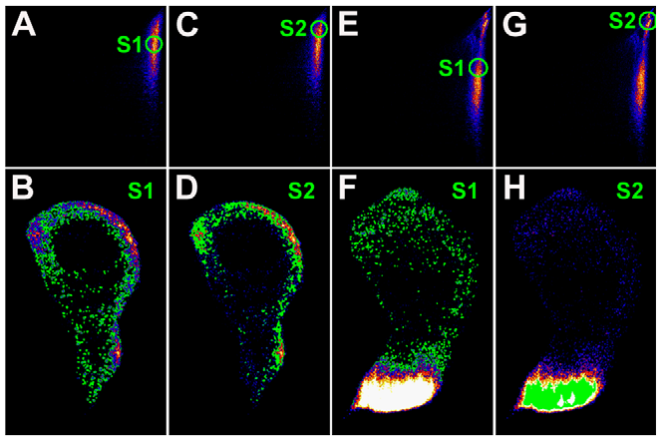
Colocalization analysis highlights the *pannier* domain when copper transport genes are manipulated. Localization of correlation regions derived from colocalization analyses in Figure 4. Splines (green circles in A, C, E, G) were drawn around specific regions of Cu-Zn colocalization clusters for a wild-type wing imaginal disc (A, C) and a *pannier-GAL4*/*UAS dNCtr1A*+*UAS Ctr1B* (E, G) disc. The GeoPIXE software then displays the location of these data points as green spots superimposed on the copper distribution map of the tissue analysed (B, D, F, H). A spline positioned at a moderate relative copper level (A) reveals pixels spread evenly across the wild-type disc (B). A spline positioned at a high relative copper level (C) reveals clustering at the margins of the wild-type disc (D). On a *pannier-GAL4*/*UAS dNCtr1A*+*UAS Ctr1B* disc, a spline positioned at a moderate relative copper level (E) also reveals an even spread of pixels (F) whereas a spline positioned at the high relative-copper cluster highlights exclusively the dorsal *pannier* domain of the disc where the genetic manipulations are targeted to.

To visualise where in the tissue selected correlation families lie, splines (green circles in Figure 5A, C, E and G) were drawn at different parts of the colocalization scatter plots. The specific pixels corresponding to these data points are then represented as green dots on the copper XRF images shown directly below each scatter plot (Figure 5B, D, F and H). Figures 5A and C are the same Cu-Zn wildtype disc elemental association scatter plot shown in Figure 4A, with splines placed at moderate (Figure 5A, S1) and high (Figure 5C, S2) relative copper levels. The moderate relative copper levels are distributed throughout the imaginal disc (Figure 5B, S1) whereas the high relative copper levels are clustered more at the margins of the disc (Figure 5D, S2), confirming our visual interpretation that, relative to zinc, copper levels are elevated at the disc margins. When the same analysis is performed on the bimodal Cu-Zn colocalization scatter plot from a *pannier-GAL4*/*UAS dNCtr1A-FLAG*+*UAS Ctr1B-FLAG* wing disc (Figure 4D, 5E–H), the moderate relative copper levels are again distributed throughout the disc, whereas the high relative copper levels are now concentrated solely in the dorsal *pannier* domain where copper uptake has been genetically induced. Again, this analysis confirms our visual interpretation of the original copper XRF image. Therefore Pearson colocalization analysis can be used effectively to identify specific regions of altered element association in an unbiased manner.

### No decrease in copper levels is detectable under decreased uptake/increased efflux conditions in the wing imaginal discs

**Figure 6:**
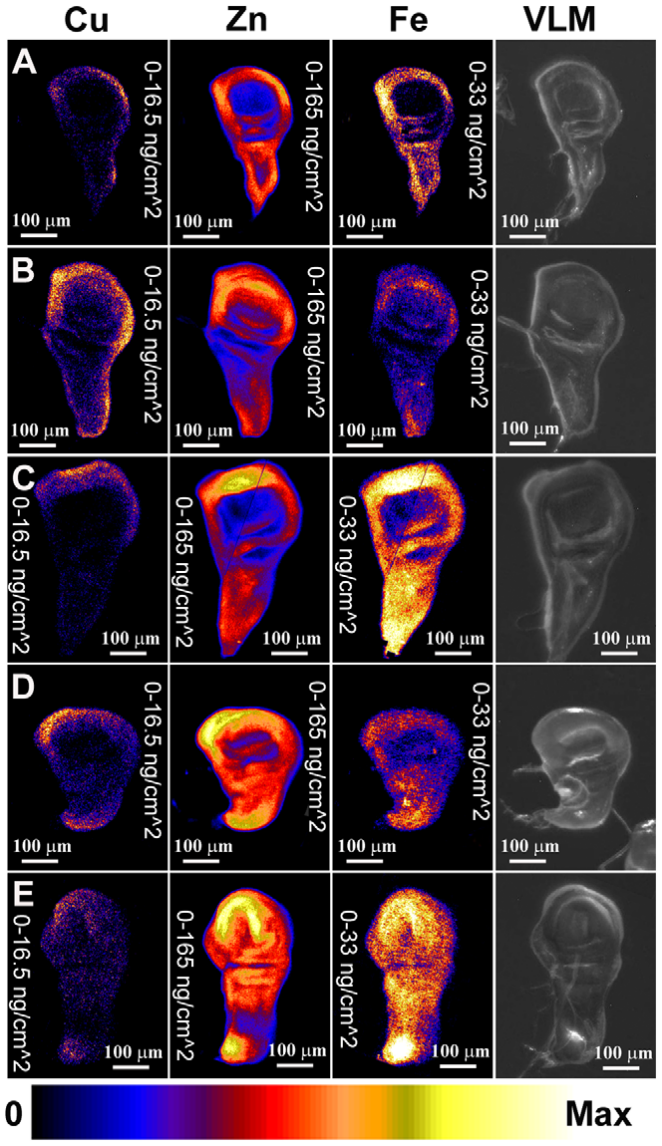
No decrease in copper is observed upon decreased uptake/increased efflux in wing discs. XRF analysis of third instar wing imaginal discs, showing distribution of copper (left panel), zinc (middle panel) and iron (right panel). Far-right panel shows a visible light microscopy (VLM) image of each disc. A) Wild-type control disc. B) One copy of *Ctr1A* RNAi transgene under *pannier-GAL4* control. C) One copy of *DmATP7-FLAG* transgene under *pannier-GAL4* control. D) One copy of *DmATP7* RNAi transgene under *pannier-GAL4* control. E) One copy of *DmATP7 DN-FLAG* (dominant negative) transgene. In each experimental case (B–E), there is no change in copper distribution relative to zinc.

Having determined that XRF can detect increased copper levels, the next question was whether decreased copper levels can also be detected. Suppression of *Ctr1A* alone or in combination with *Ctr1B* suppression results in loss of thoracic cuticle pigmentation and in extreme cases, clefting of the adult thorax and a reduced, flattened adult eye [20]. Over expression of *DmATP7* similarly causes thoracic hypopigmentation, sensory bristle loss and thoracic clefting [15]. These phenotypes are thought to be due to insufficient cellular copper levels caused by reduced copper uptake or increased copper efflux respectively. However analysis of several of these genotypes of varying severity revealed no obvious decrease in copper levels in the *pannier* domain, with these imaginal discs appearing indistinguishable from wild-type imaginal discs (Figure 6A–E). Elemental maps of additional wing discs of the same genotypes seen in Figure 6 are shown in Figure S3 for comparison.

### Elemental analysis of adult *Drosophila* head sections reveals differences in relative metal levels between eye and brain tissue

**Figure 7:**
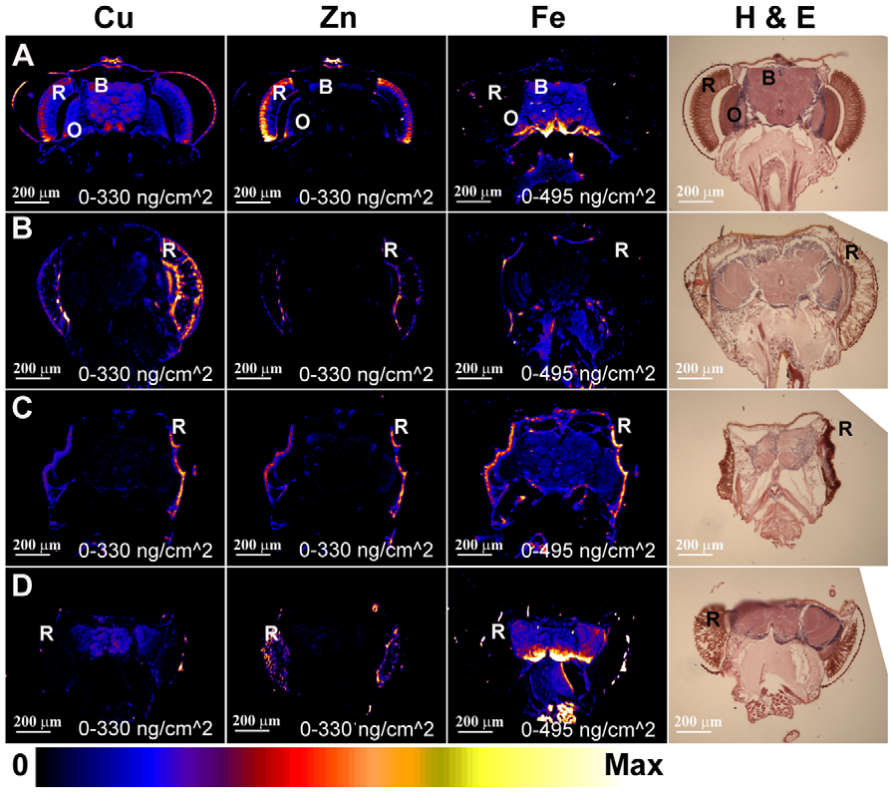
XRF analysis of *Drosophila* adult head sections demonstrates both increased and decreased copper levels. XRF analysis of adult *Drosophila* head sections, showing distribution of copper (left panel), zinc (middle panel) and iron (right panel). Far-right panel shows an adjacent section stained with haemotoxylin-eosin (H & E). R denotes Retina, O denotes Optic Nerve and B denotes Brain. Element quantification is normalised so that maximum values are as follows: copper - 330 ng/cm^2^, zinc – 330 ng/cm^2^, iron - 495 ng/cm^2^. A) Wild-type control sections. B) One copy of *Ctr1B-FLAG* transgene under *GMR-GAL4* control. Copper is increased in the retina relative to the brain in comparison to wild-type. C) One copy of *Ctr1A* RNAi transgene under *GMR-GAL4* control. The retina is collapsed into a single fused layer of tissue rich in zinc, copper and iron. D) One copy of *DmATP7-FLAG* transgene under *GMR-GAL4* control. Copper is decreased in the retina relative to the brain in comparison to wild-type.

Since the copper-related phenotypes described in the introduction are all adult phenotypes, adult head sections were subjected to elemental analysis by XRF to determine if changes in copper levels could be detected. Fly head sections are well characterized, with typical eye and brain features easily distinguishable. Furthermore using the *GMR-GAL4* driver, genetic manipulations can be targeted to the retinal tissue of the eye while the remaining optic nerve and brain tissue is left unaffected. XRF analysis of wild-type fly head sections revealed that, unlike the larval imaginal discs, elemental distribution varied strikingly between retinal and brain tissues. Copper levels appear to be consistent across both the retina and brain (R and B, Figure 7A). Zinc levels, in contrast, are higher in the retinal tissue relative to the brain (Figure 7A). Iron shows a complementary distribution to zinc, with relatively high levels in the brain compared to retina (Figure 7A). This differential distribution pattern was consistent across all wild-type head sections analysed (n = 4).

Next, the effect of increased copper uptake was examined. The *UAS Ctr1B-FLAG* transgene gives a mild rough-eye phenotype when expressed in the developing eye under *GMR-GAL4* control [20]. Examination of adult head sections from *GMR-GAL4*/*UAS Ctr1B-FLAG* flies showed a shift in copper distribution – copper levels were higher in the retina than in the brain in contrast to the wild-type situation (Figure 7B, n = 3). Zinc and iron distribution, on the other hand, remained the same as in the wild-type situation (Figure 7B). Thus an increase in copper levels specifically in the eye tissue can be detected by XRF.

To determine whether a decrease in copper levels can be detected, adult head sections were analysed from flies in which *Ctr1A* had been suppressed in the retina. These flies show a reduced, flattened eye with abnormal morphology [20]. XRF analysis of these heads revealed a severely disrupted eye morphology where the eye tissue had collapsed and could not be distinguished from the head cuticle and the brain (Figure 7C, n = 3). A single layer of tissue enriched in copper, zinc and iron was all that remained of the normal eye tissue (Figure 7C). Therefore these eyes were so badly compromised that no information regarding copper levels in the eye tissue could be obtained.

A reduction in copper levels can also be obtained by increased efflux caused by *DmATP7* over expression. This causes a typical copper depletion phenotype in the adult thorax/abdomen but causes no visible defects in adult eye morphology [20]. Adult head sections from flies with a *GMR-GAL4*/*UAS DmATP7-FLAG* genotype were analysed by XRF. In contrast to wild-type, reduced copper levels were seen in the eyes relative to the brain tissue (Figure 7D, n = 3). Zinc and iron distribution remained normal. Elemental maps of additional adult head sections of the same genotypes seen in Figure 7 are shown in Figure S4 for comparison.

## Discussion

The results presented here demonstrate that XRF can be used to rapidly and reliably assess elemental levels in two different *Drosophila* tissues, the larval imaginal discs and adult eye/head sections. All three elements analysed, copper, zinc and iron, showed an even distribution throughout the wing imaginal disc, although higher copper levels were often seen at the ventral margin of the disc. As these imaginal discs are still growing at the time of dissection, one possibility is that copper is distributed to the outer edge of growing tissues, reminiscent of the active efflux of copper observed at the tips of nascent endothelial cell filopodia undergoing angiogenesis [37], [38]. The functional significance of this remains to be determined however it is worth noting that extreme copper depletion causes a thoracic cleft in *Drosophila*. The fly thorax is formed from the dorsal region of the two wing imaginal discs. During pupariation, these epithelial sheets spread and extend towards each other, eventually fusing in a process similar to *Drosophila* embryonic dorsal closure. Possibly, copper is required for these morphogenetic movements.

By increasing copper uptake (*dNCtr1A*+*Ctr1B*) or increasing uptake and blocking efflux (*dNCtr1A*+*DmATP7* RNAi), clear increases in copper levels were observed by XRF in the *pannier* domain relative to the remainder of the wing imaginal disc, demonstrating that XRF is an effective method for assessing changes in elemental levels. However increases in copper were only seen with genotypes that cause clear phenotypic defects in the fly [20]. For instance even two copies of the *dNCtr1A-FLAG* construct have no effect on copper levels nor do they cause any phenotypic defect in the fly eye or thorax, despite this transgene having demonstrable copper uptake activity [15]. Presumably the *dNCtr1A-FLAG* over expressing cells are able to compensate for the increased copper uptake by increasing copper efflux. Indeed, blocking copper efflux (*DmATP7* RNAi) in the presence of *dNCtr1A-FLAG* over expression causes dramatic increases in copper levels (this work) and strong phenotypic defects [20].

The absence of any detectible change in copper levels under *dNCtr1A-FLAG* expression alone stands in contrast to results from cultured cells where ectopic expression of *Ctr1A* or simply increasing media copper levels causes a clear increase in copper cellular levels [14], [39]. The results presented here suggest that cells within tissues may behave differently to isolated, cultured cells. Imaginal disc cells appear to be able to maintain stable copper levels until pushed to a certain threshold, beyond which homeostasis is strongly disrupted and copper levels increase dramatically, with clearly deleterious results as evidenced by the eye and thorax phenotypes observed [20], [21]. This ability to maintain stable copper levels may vary with cell type. Enterocytes, for instance, could be considerably more flexible in their copper content due to their role in dietary copper absorption. This flexibility may be provided by metallothionein sequestration. Interestingly *metallothionein* genes are strongly expressed in *Drosophila* enterocytes but not in imaginal disc cells [12].

In contrast to the imaginal discs, the adult eye/head sections showed a more complex metal distribution pattern. While copper levels were even across the entire section, zinc levels were clearly elevated in the eye tissue relative to the brain and iron showed a complementary pattern, with high levels in the brain relative to the eye tissue. Given that all three metals are known to be abundant and necessary in neuronal tissue, the complementary distribution of zinc and iron was somewhat unexpected. Possibly zinc is required at very high levels for photoreception in the eye tissue and iron levels are decreased as a result. It would be fascinating to see if manipulating iron or zinc levels in the eye tissue had a compensatory effect on the other metal.

As expected, ectopic *Ctr1B* expression in the eye caused a clear increase in copper levels in this tissue relative to the brain. Unfortunately eyes in which *Ctr1A* had been suppressed could not be assessed for copper content due to strong morphological deformities. However, eyes with *DmATP7* over expression, which causes no eye phenotype but a strong thorax phenotype [15], [20], showed clear decrease in copper levels. Therefore in adult tissues at least, copper redistribution is clearly visualised using XFM.

No decrease in copper levels in the *pannier* domain was observed in wing imaginal discs with genotypes known to cause clear copper deficiency phenotypes in adult flies. One possibility is that even a mild, undetectable decrease in cellular copper levels is sufficient to cause dramatic adult defects. Alternatively, since the larval stage studied is approximately five days before the adult fly emerges, imaginal disc cells may be able to retain appropriate copper levels up to a point after the late larval stage examined here. This is more likely given that decrease in copper can be seen in adult eye/head sections with genotypes where no such decrease was observed in the imaginal discs.

The results presented here demonstrate that XFM has the potential to be used as a rapid and reliable technique for elemental analysis not only in *Drosophila* tissues but many other experimental systems. A previous restriction in such work has been the long scan times required to compile sufficient data. This has limited the number of biological replicates that can be assessed in a reasonable time. With the 96-element prototype Maia detector used here, single wing imaginal discs could be scanned in under 30 minutes. The new 384-element Maia detector recently installed at the Australian Synchrotron XFM beamline has increased detector solid angle and a detector geometry that allows essentially unlimited lateral scanning of samples [40]. The increased detection efficiency together with the ability to mount and scan numerous samples in automated batch mode will allow the analysis of over 50 samples in a 24 hour period, further enhancing the statistical power of this technique. With these improvements, XFM becomes a sensitive and robust method to rapidly measure tissue elemental levels and carry out more sophisticated analyses of metal homeostasis in whole tissues and even whole organisms.

## Supporting Information

Figure S1 **Additional XRF images of wing imaginal discs of the same genotypes as** **Figure 2** **.** XRF analysis of third instar wing imaginal discs, showing distribution of copper (left panel), zinc (middle panel) and iron (right panel). Distribution is shown as a heat map with the relative concentration shown at the bottom of the figure. A and B) Wild-type control discs. C to F) One copy (C, D) or two copies (E, F) of *dNCtr1A-FLAG* under *pannier-GAL4* control. No change in copper distribution relative to zinc is seen. G to I) One copy of *dNCtr1A-FLAG* together with one copy of *Ctr1B-FLAG* under *pannier-GAL4* control. Dramatic increase in copper relative to zinc is seen in the dorsal *pannier* domain (arrows). J) One copy of *dNCtr1A-FLAG* together with one copy of *DmATP7* RNAi under *pannier-GAL4* control. Strong increase in copper relative to zinc is seen in the dorsal *pannier* domain (arrow).
(TIF)

Figure S2 **Additional XRF images of eye imaginal discs of the same genotypes as** **Figure 3** **.** XRF analysis of third instar eye imaginal discs, showing distribution of copper (left panel), zinc (middle panel) and iron (right panel). Distribution is shown as a heat map with the relative concentration shown at the bottom of the figure. A) Wild-type control discs. B) One copy of *dNCtr1A-FLAG* together with one copy of *Ctr1B-FLAG* under *pannier-GAL4* control. Dramatic increase in copper relative to zinc is seen in the dorsal *pannier* domain (arrow).
(TIF)

Figure S3 **Additional XRF images of wing imaginal discs of the same genotypes as** **Figure 6** **.** XRF analysis of third instar wing imaginal discs, showing distribution of copper (left panel), zinc (middle panel) and iron (right panel). Distribution is shown as a heat map with the relative concentration shown at the bottom of the figure. A) One copy of *Ctr1A* RNAi transgene under *pannier-GAL4* control. B) One copy of *DmATP7-FLAG* transgene under *pannier-GAL4* control. C) One copy of *DmATP7* RNAi transgene under *pannier-GAL4* control. D) One copy of *DmATP7 DN-FLAG* (dominant negative) transgene. In each experimental case (A–D), there is no change in copper distribution relative to zinc.
(TIF)

Figure S4 **Additional XRF images of adult head sections of the same genotypes as** **Figure 7** **.** XRF analysis of adult *Drosophila* head sections, showing distribution of copper (left panel), zinc (middle panel) and iron (right panel). A) Wild-type control sections. B) One copy of *Ctr1B-FLAG* transgene under *GMR-GAL4* control. Copper is increased in the retina relative to the brain in comparison to wild-type. C) One copy of *Ctr1A* RNAi transgene under *GMR-GAL4* control. The retina is collapsed into a single fused layer of tissue rich in zinc, copper and iron. D) One copy of *DmATP7-FLAG* transgene under *GMR-GAL4* control. Copper is decreased in the retina relative to the brain in comparison to wild-type.
(TIF)
