# Supplementary figures and images for: Detection of Genetically Altered Copper Levels in Drosophila Tissues by Synchrotron X-Ray Fluorescence Microscopy
Source: PLoS One. 2011 Oct 28;6(10):e26867. doi: 10.1371/journal.pone.0026867 (PMC3203902; doi:10.1371/journal.pone.0026867)

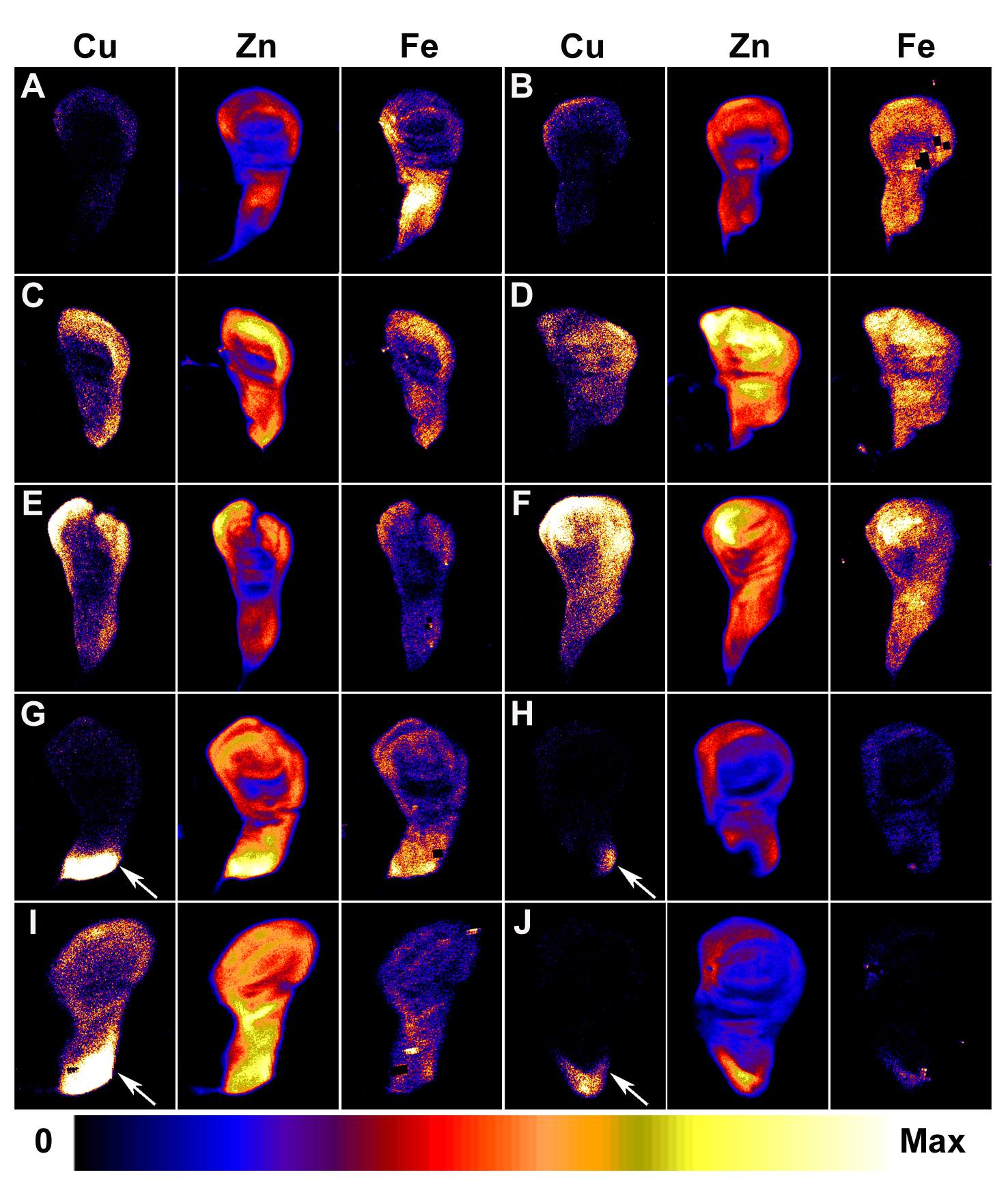

Supplement: Figure S1 — Additional XRF images of wing imaginal discs of the same genotypes as Figure 2 . XRF analysis of third instar wing imaginal discs, showing distribution of copper (left panel), zinc (middle panel) and iron (right panel). Distribution is shown as a heat map with the relative concentration shown at the bottom of the figure. A and B) Wild-type control discs. C to F) One copy (C, D) or two copies (E, F) of dNCtr1A-FLAG under pannier-GAL4 control. No change in copper distribution relative to zinc is seen. G to I) One copy of dNCtr1A-FLAG together with one copy of Ctr1B-FLAG under pannier-GAL4 control. Dramatic increase in copper relative to zinc is seen in the dorsal pannier domain (arrows). J) One copy of dNCtr1A-FLAG together with one copy of DmATP7 RNAi under pannier-GAL4 control. Strong increase in copper relative to zinc is seen in the dorsal pannier domain (arrow). (TIF) [file pone.0026867.s001.tif]

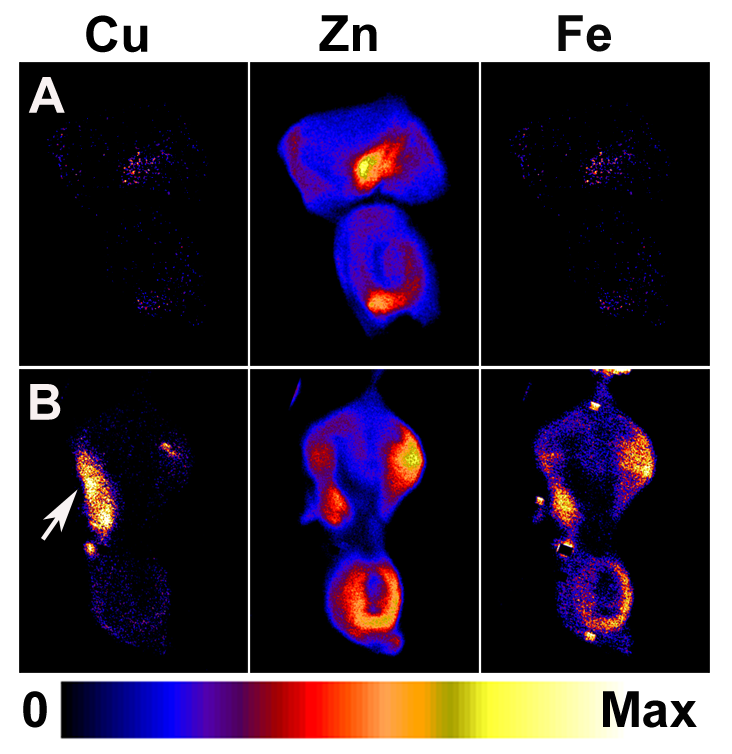

Supplement: Figure S2 — Additional XRF images of eye imaginal discs of the same genotypes as Figure 3 . XRF analysis of third instar eye imaginal discs, showing distribution of copper (left panel), zinc (middle panel) and iron (right panel). Distribution is shown as a heat map with the relative concentration shown at the bottom of the figure. A) Wild-type control discs. B) One copy of dNCtr1A-FLAG together with one copy of Ctr1B-FLAG under pannier-GAL4 control. Dramatic increase in copper relative to zinc is seen in the dorsal pannier domain (arrow). (TIF) [file pone.0026867.s002.tif]

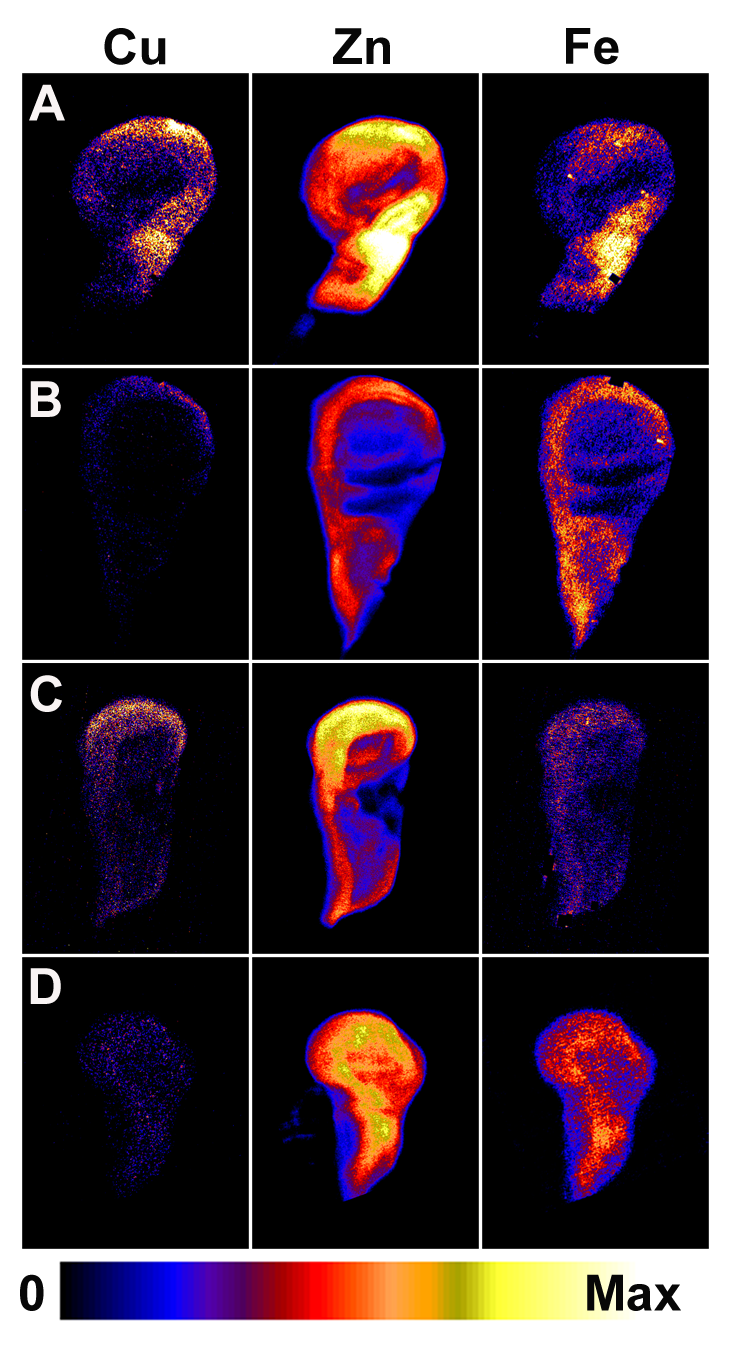

Supplement: Figure S3 — Additional XRF images of wing imaginal discs of the same genotypes as Figure 6 . XRF analysis of third instar wing imaginal discs, showing distribution of copper (left panel), zinc (middle panel) and iron (right panel). Distribution is shown as a heat map with the relative concentration shown at the bottom of the figure. A) One copy of Ctr1A RNAi transgene under pannier-GAL4 control. B) One copy of DmATP7-FLAG transgene under pannier-GAL4 control. C) One copy of DmATP7 RNAi transgene under pannier-GAL4 control. D) One copy of DmATP7 DN-FLAG (dominant negative) transgene. In each experimental case (A–D), there is no change in copper distribution relative to zinc. (TIF) [file pone.0026867.s003.tif]

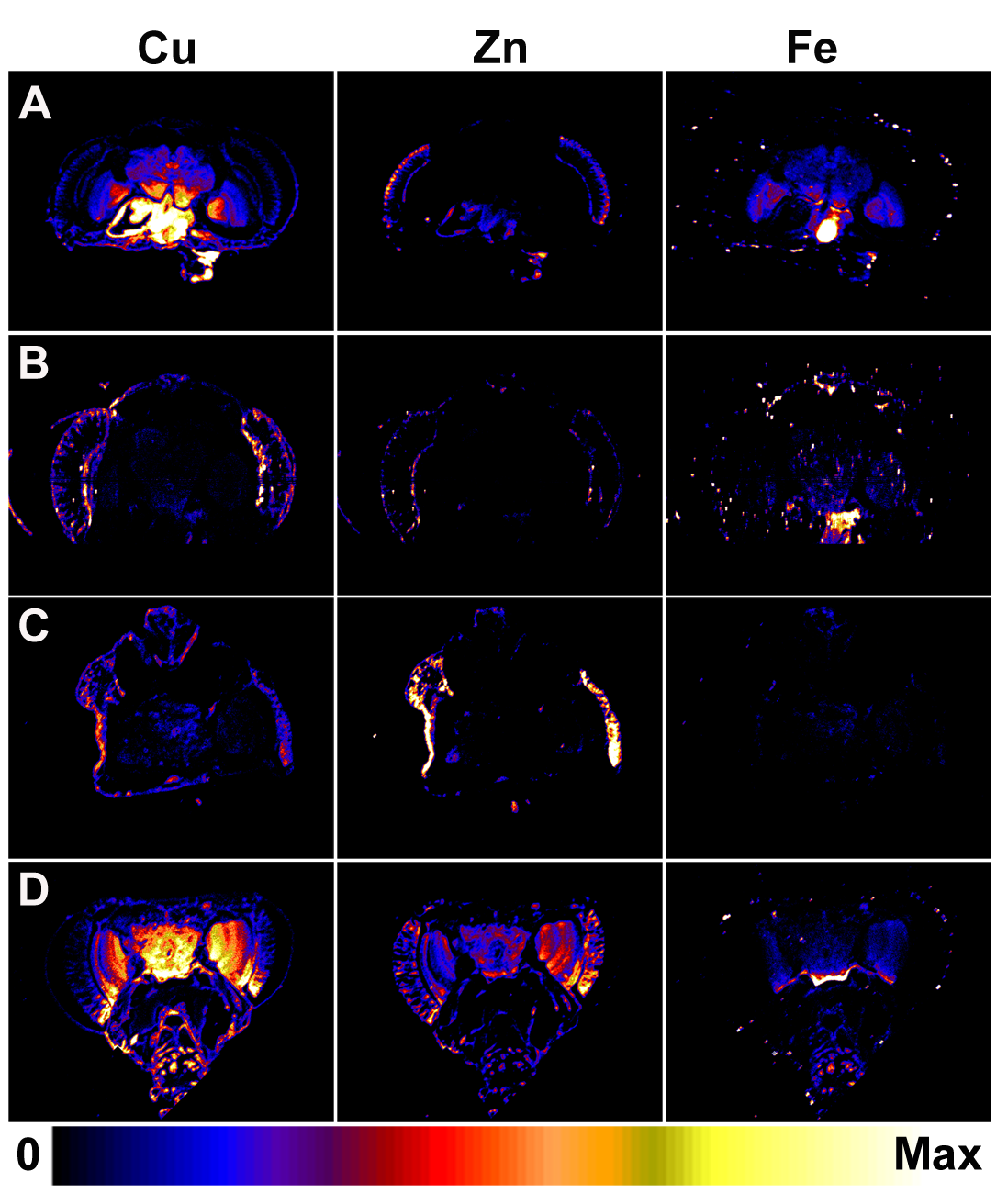

Supplement: Figure S4 — Additional XRF images of adult head sections of the same genotypes as Figure 7 . XRF analysis of adult Drosophila head sections, showing distribution of copper (left panel), zinc (middle panel) and iron (right panel). A) Wild-type control sections. B) One copy of Ctr1B-FLAG transgene under GMR-GAL4 control. Copper is increased in the retina relative to the brain in comparison to wild-type. C) One copy of Ctr1A RNAi transgene under GMR-GAL4 control. The retina is collapsed into a single fused layer of tissue rich in zinc, copper and iron. D) One copy of DmATP7-FLAG transgene under GMR-GAL4 control. Copper is decreased in the retina relative to the brain in comparison to wild-type. (TIF) [file pone.0026867.s004.tif]
